# Targeting the endocannabinoid/CB1 receptor system for treating obesity in Prader–Willi syndrome

**DOI:** 10.1016/j.molmet.2016.10.004

**Published:** 2016-10-22

**Authors:** Ibrahim Knani, Brian J. Earley, Shiran Udi, Alina Nemirovski, Rivka Hadar, Asaad Gammal, Resat Cinar, Harry J. Hirsch, Yehuda Pollak, Itai Gross, Talia Eldar-Geva, Daniela P. Reyes-Capo, Joan C. Han, Andrea M. Haqq, Varda Gross-Tsur, Rachel Wevrick, Joseph Tam

**Affiliations:** 1 Obesity and Metabolism Laboratory, The Institute for Drug Research, School of Pharmacy, Faculty of Medicine, The Hebrew University of Jerusalem, Jerusalem, Israel; 2 Laboratory of Physiologic Studies, National Institute on Alcohol Abuse and Alcoholism, Bethesda, MD, USA; 3 Neuropediatric Unit, Department of Pediatrics, Shaare Zedek Medical Center, Jerusalem, Israel; 4 Reproductive Endocrinology and Genetics Unit, Department of Obstetrics and Gynecology, Shaare Zedek Medical Center, Jerusalem, Israel; 5 Unit on Metabolism and Neuroendocrinology, Eunice Kennedy Shriver National Institute of Child Health and Human Development, Bethesda, MD, USA; 6 Department of Pediatrics, University of Tennessee Health Science Center, Children’s Foundation Research Institute, Le Bonheur Children's Hospital, Memphis, TN, USA; 7 Department of Physiology, University of Tennessee Health Science Center, Memphis, TN, USA; 8 Department of Pediatrics, University of Alberta, Edmonton, AB, Canada; 9 Department of Medical Genetics, University of Alberta, Edmonton, AB Canada

**Keywords:** Endocannabinoids, PWS, Magel2, Peripheral CB1 blockade, Metabolic syndrome

## Abstract

**Objective:**

Extreme obesity is a core phenotypic feature of Prader–Willi syndrome (PWS). Among numerous metabolic regulators, the endocannabinoid (eCB) system is critically involved in controlling feeding, body weight, and energy metabolism, and a globally acting cannabinoid-1 receptor (CB_1_R) blockade reverses obesity both in animals and humans. The first-in-class CB_1_R antagonist rimonabant proved effective in inducing weight loss in adults with PWS. However, it is no longer available for clinical use because of its centrally mediated, neuropsychiatric, adverse effects.

**Methods:**

We studied eCB ‘tone’ in individuals with PWS and in the *Magel2*-null mouse model that recapitulates the major metabolic phenotypes of PWS and determined the efficacy of a peripherally restricted CB_1_R antagonist, JD5037 in treating obesity in these mice.

**Results:**

Individuals with PWS had elevated circulating levels of 2-arachidonoylglycerol and its endogenous precursor and breakdown ligand, arachidonic acid. Increased hypothalamic eCB ‘tone’, manifested by increased eCBs and upregulated CB_1_R, was associated with increased fat mass, reduced energy expenditure, and decreased voluntary activity in *Magel2*-null mice. Daily chronic treatment of obese *Magel2*-null mice and their littermate wild-type controls with JD5037 (3 mg/kg/d for 28 days) reduced body weight, reversed hyperphagia, and improved metabolic parameters related to their obese phenotype.

**Conclusions:**

Dysregulation of the eCB/CB_1_R system may contribute to hyperphagia and obesity in *Magel2*-null mice and in individuals with PWS. Our results demonstrate that treatment with peripherally restricted CB_1_R antagonists may be an effective strategy for the management of severe obesity in PWS.

## Highlights

• Circulating levels of endocannabinoids are increased in individuals with PWS.
• *Magel2* is a negative regulator of hypothalamic endocannabinoid ‘tone’.
• Peripherally-restricted CB1 receptor blockade reverses obesity in *Magel2*-null mice.

## 1 Introduction

Prader–Willi syndrome (PWS) is a complex neurogenetic disorder caused by the loss of a paternally inherited, imprinted cluster of genes at human chromosome 15q11-q13. PWS is characterized by childhood-onset hyperphagia-associated obesity, intellectual disability, short stature, hypogonadism, and disturbances of sleep and thermoregulation [1]. The endogenous mechanisms that control appetite and consequently lead to the development of obesity in individuals with PWS are unclear. In particular, a direct link between either genetic or hormonal dysfunction related to PWS and feeding pathways in the hypothalamus in humans has yet to be established.

Among the numerous hypothalamic appetite regulators, endocannabinoids (eCBs) are critically involved in regulating appetite, body weight, and metabolism. The main eCBs, arachaidonoyl ethanolamide (anandamide; AEA) and 2-arachidonoylglycerol (2-AG), are lipid-like signaling mediators that interact with a cell surface cannabinoid-1 receptor (CB_1_R) [2]. Both eCBs are generated 'on demand' in the cell membrane from phospholipid precursors, such as arachidonic acid (AA). Whereas activation of CB_1_R by eCBs promotes appetite via a leptin-regulated hypothalamic neural appetitive circuitry [3], its chronic blockade by globally acting CB_1_R antagonists inhibits food intake both in rodents [4], [5], [6], [7] and individuals with obesity [8], [9], [10], [11]. Interestingly, the first-in-class CB_1_R antagonist rimonabant was proved effective in reducing body weight, fat mass, and leptin levels in adults with PWS [12]. However, because of neuropsychiatric side effects such as anxiety, dysthymia, and paranoia and the increased susceptibility to psychiatric disorders, the use of rimonabant is no recommended in PWS.

A role for peripheral eCBs in regulating appetite and body weight is indicated by the increased peripheral eCB ‘tone’ in diet-induced obesity (DIO), manifested by increased expression of CB_1_R in peripheral tissues [13], [14], [15], [16], [17] and/or elevated levels of AEA and 2-AG [18], [19]. Therefore, peripherally restricted CB_1_R antagonists may also represent a novel approach to reduce appetite and body weight. Indeed, our recent findings in DIO mice suggest that peripheral CB_1_R blockade is as efficacious as globally acting CB_1_R antagonism in reducing appetite and body weight without causing behavioral effects that are associated with blocking the receptor in the central nervous system (CNS) [20], [21]. This suggests that these compounds have excellent therapeutic potential for treating obesity and its metabolic complications. Here, we assessed to what extent the eCB/CB_1_R system is involved in the pathogenesis of obesity in PWS both in humans and mice. We demonstrate that a novel, peripherally restricted CB_1_R antagonist, JD5037, is effective in reversing obesity and its deleterious metabolic effects in DIO *Magel2*-null mice, which exhibit fundamental aspects of the PWS phenotype [22].

## 2 Materials and methods

### 2.1 Subjects

#### 2.1.1 Israeli cohort

The study protocol was approved by the Institutional Review Board of Shaare Zedek Medical Center. Written informed consent was obtained from each of the participants. Subjects with genetically confirmed PWS were recruited from the Israeli National Multidisciplinary PWS Clinic at Shaare Zedek Medical Center, Jerusalem. The control group was comprised of volunteers: 9 with body mass index (BMI) <25 kg/m^2^ (6 males/3 females) and 24 overweight or obese (11 males/13 females) with BMI >25 kg/m^2^. Subjects with chronic illnesses, uncontrolled hypothyroidism, diabetes mellitus, or exposure to hormone medication including estrogen-containing compounds, glucocorticoids, or androgens were excluded. Venous whole blood was collected from subjects, separated to plasma, and stored at −80 °C until further assessed. The Israeli participants underwent biochemical testing, following a 12 h fast for serum lipid profile, glucose and insulin. Measurements of leptin and adiponectin were performed by ELISAs (Phoenix, Pharmaceuticals, USA). The homeostasis model assessment insulin resistance (HOMA-IR) was calculated as fasting serum insulin ([μU/mL] X fasting plasma glucose [mmol/L]/22.5). Height and weight were measured and the BMI was calculated.

#### 2.1.2 North American cohort

The study protocol was approved by the Institutional Review Boards of the National Institutes of Health and the University of Alberta. Informed written consent from the parents/guardians and assent from each child were obtained. Subjects with PWS previously confirmed by genetic testing were recruited from the pediatric endocrinology clinic at the University of Alberta and through online advertisements for obesity-related research studies at the National Institutes of Health (ClinicalTrials.gov Identifier: NCT01517048). Age-, sex-, and BMI-matched control subjects were recruited through local advertisements in the Edmonton and Washington, D.C., metropolitan areas. Subjects with diabetes mellitus, chronic severe kidney, liver or neurologic disorders, or those on concomitant investigational drug were excluded. Venous whole blood was collected from subjects and centrifuged to separate plasma, which was stored at −80 °C until further assessed. Height was measured in the morning as an average of triplicate measurements using a Harpenden stadiometer. Weight was measured in light clothing using a digital scale. Age- and sex-normed BMI-Z score were calculated [23].

### 2.2 Animals and experimental protocol

The experimental protocol used was approved by the Institutional Animal Care and Use Committee of the Hebrew University, which is an AAALAC International accredited institute. The *Magel2*-null mice (C57BL/6-Magel2tm1Stw/J, Jackson Laboratory stock no. 009062) were maintained on a C57Bl/6J background for at least 15 generations and were genotyped as previously described [22], [24]. Mice carrying a paternally inherited lacZ-knockin allele were functionally null for *Magel2* and were referred to as *Magel2*-null; littermates that were wild-type for *Magel2* were used as controls. Both male and female mice from 6 to 22 weeks of age were used and maintained under a 12-h light/dark cycle and were fed *ad libitum*. To generate DIO mice (body weight >42 g), both genotypes were fed either a high-fat diet (HFD) (Research Diet, D12492; 60% of calories from fat, 20% from protein, and 20% from carbohydrates) or a standard diet (STD, NIH-31 rodent diet) for 12 weeks. During the HFD or STD feeding, body weight was recorded once a week, and body composition was assessed by EchoMRI-100 (Echo Medical Systems LLC, Houston, TX) every four weeks. Once the animals became obese, they were treated chronically (28 d) with vehicle (V; 1% Tween80, 4% DMSO, 95% Saline), JD5037 (J), or SLV319 (S) at a dose of 3 mg/kg, i.p. Age-matched control mice on STD were treated with V. During the treatment period, body weight and food intake were monitored daily. Mice were euthanized by cervical dislocation under anesthesia; the brain, hypothalamus, liver, and combined fat pads were removed, weighed, and snap-frozen, and trunk blood was collected for determining the endocrine and biochemical parameters.

### 2.3 Tissue levels of JD5037

Mice received a single dose (3 mg/kg, i.p.) of JD5037 and were euthanized 1 h later. Blood was collected, and the mice were perfused with phosphate-buffered saline (PBS) for 1 min to remove the drug from the intravascular space before removing the brain and liver. Tissues and serum were extracted, and drug levels were determined by liquid chromatography/tandem mass spectrometry (LC–MS/MS) as described previously [21], with modifications. The analyses were conducted on a TSQ Quantum Access Max triple quadrupole mass spectrometer (Thermo Scientific, San Jose, CA, USA) coupled with an UHPLC system, which included a Dionex Pump and an Accela Autosampler (Thermo Scientific, San Jose, CA, USA). A Kinetex™ (Phenomenex, Torrance, CA, USA) column (C18, 2.6 μm particle size, 100A pore size, 50 × 2.1 mm) was used for separation. Gradient elution mobile phases consisted of 0.1% formic acid in water (phase A) and 0.1% formic acid in acetonitrile (phase B). Gradient elution (0.25 mL/min) was held at 20% B for 0.7 min, followed by a linear increase to 80% B for 0.8 min, and maintained at 80% B for 5.5 min, then increased linearly to 95% B for 0.3 min, and maintained at 95% B for 2.7 min. JD5037 was detected in a positive ion mode using electron spray ionization (ESI) and the multiple reaction monitoring (MRM) mode of acquisition. The mass spectrometer parameters were set as follow: spray voltage 5000 V; vaporizer temperature 350 °C; capillary temperature 250 °C; sheath and auxiliary gases 35 and 10 arbitrary units, respectively; argon was used as the collision gas.

The levels of JD5037 were analyzed using [^2^H_4_]arachidonoyl ethanolamide ([^2^H_4_]AEA) as an internal standard. The molecular ions and fragments for each compound were measured as follows: *m*/*z* 572 → 555 (quantifier) and 572 → 111 (qualifier) for JD5037 (collision energy: 17 V and 49 V, respectively) and *m*/*z* 352 → 66 (quantifier) and 352 → 91 (qualifier) for [^2^H_4_]AEA (collision energy: 30 V and 45 V, respectively). TSQ Tune Software (Thermo Scientific, San Jose, CA, USA) was used to optimize the tuning parameters. Data acquisition and processing were carried out using the Xcalibur program (Thermo Scientific, San Jose, CA, USA). The amounts of JD5037 in the samples were determined against a standard curve. Values are expressed as ng/g or ng/mL in wet tissue weight or serum volume, respectively.

### 2.4 Endocannabinoid measurements

AEA, 2-AG, and AA in serum, plasma, and tissues were extracted, purified, and quantified by the stable isotope dilution LC–MS/MS method as described previously [21] with the following modifications. For serum and plasma, total proteins were precipitated with ice-cold acetone/Tris buffer (50 mM, pH 8.0). Then, samples were homogenized in 0.5 mL of ice-cold methanol/Tris buffer (50 mM, pH 8.0), 1:1, containing [^2^H_4_]AEA as an internal standard. Homogenates were then extracted with CHCl_3_:MeOH (2:1, vol/vol), and washed three times with ice-cold CHCl_3_, dried under nitrogen flow, and reconstituted with MeOH. For fat pads and cerebral cortex, samples were first homogenized, extracted, and washed as described above. Then, total proteins were precipitated. For hypothalami, samples from free-fed male and female mice were first homogenized in Tris buffer, protein content was determined, and extraction continued as for fat pads and brains.

LC–MS/MS analyses were conducted on an AB Sciex (Framingham, MA, USA) Triple Quad™ 5500 mass spectrometer coupled with a Shimadzu (Kyoto, Japan) UHPLC System. Liquid chromatographic separation was obtained using a Kinetex™ (Phenomenex) column (C18, 2.6 μm particle size, 100 × 2.1 mm). eCBs were detected in a positive ion mode under ESI and MRM conditions. The molecular ions and fragments for each compound were: *m*/*z* 348.3 → 62.1 (quantifier) and 91.1 (qualifier) for AEA, *m*/*z* 379.3 → 91.1 (quantifier) and 287.3 (qualifier) for 2-AG, *m*/*z* 305.2 → 91.1 (quantifier) and 77.1 (qualifier) for AA and *m*/*z* 352.3 → 66.1 (quantifier) and 91.1 (qualifier) for [^2^H_4_]AEA. The levels of AEA, 2-AG, and AA in the samples were measured against standard curves. The values are expressed as fmol/mL, pmol/mL, or nmol/mL in serum and plasma, fmol/mg, pmol/mg, or nmol/mg in fat pad, fmol/mg protein, or pmol/mg protein in the hypothalamus.

### 2.5 Multi-parameter metabolic assessment

Metabolic and activity profiles of the mice were measured by using the Promethion High-Definition Behavioral Phenotyping System (Sable Instruments, Inc., Las Vegas, NV, USA). Data acquisition and instrument control were performed using MetaScreen software version 2.2.18.0, and the obtained raw data were processed using ExpeData version 1.8.4 using an analysis script detailing all aspects of data transformation. Mice with a free access to food and water were subjected to a standard 12 h dark/12 h dark cycle, which consisted of a 24 h acclimation period followed by a 48 h sampling duration. No major changes in body weight were documented during the acclimation period (Males: −1.6 ± 0.4 vs. −0.9 ± 0.2; Females: −0.6 ± 0.2 vs. −0.4 ± 0.1, wild-type vs. *Magel2*-null mice, respectively), and acquisition period (Males: 0.1 ± 0.2 vs. 0.2 ± 0.2; Females: −0.2 ± 0.1 vs. 0.1 ± 0.04, wild-type vs. *Magel2*-null mice, respectively). Respiratory gases were measured by the GA-3 gas analyzer (Sable Systems, Inc., Las Vegas, NV, USA) using a pull-mode, negative-pressure system. Air flow was measured and controlled by FR-8 (Sable Systems, Inc., Las Vegas, NV, USA), with a set flow rate of 2000 mL/min. Water vapor was continuously measured, and its dilution effect on O_2_ and CO_2_ was mathematically compensated. Effective mass was calculated by ANCOVA analysis as described previously [25], using the calculations described in Supplementary Figure 1. Respiratory quotient (RQ) was calculated as the ratio between CO_2_ produced to O_2_ consumed, and total energy expenditure (TEE) was calculated as *VO*_*2*_ *x* (*3*.*815* + *1*.*232* × *RQ*), normalized to effective body mass, and expressed as kcal/h/kg^eff.Mass^. Fat oxidation (FO) and carbohydrate oxidation (CHO) were calculated as *FO* = *1.69* × *VO*_*2*_ – *1*.*69* × *VCO*_*2*_ and *CHO* = *4.57* × *VCO*_*2*_ – *3*.*23* × *VO*_*2*_ and expressed as g/d/kg^eff.Mass^. Ambulatory and voluntary activities and animal positions were monitored simultaneously by collecting the calorimetry data using the XYZ beam arrays with a beam spacing of 0.25 cm and with wheels running inside the cage.

### 2.6 Real-time PCR

Total mRNA from mouse hypothalamus, cerebral cortex, and fat pads was extracted using Bio-Tri RNA lysis buffer (Bio-Lab, Israel) or the NucleoSpin^®^ RNA extraction kit (MACHEREY-NAGEL, Germany), followed by DNase I treatment (Thermo Scientific, IL, USA), and then reverse transcribed using the Iscript cDNA kit (Bio-Rad, CA). Real-time PCR was performed using the iTaq Universal SYBR Green Supermix (Bio-Rad, CA) and the CFX connect ST system (Bio-Rad, CA). The following primers for *Cnr1*: Forward, 5′-CCGCAAAGATAGTCCCAATG-3′; Reverse, 5′-AACCCCACCCAGTTTGAAC-3′ and *β-Actin*: Forward, 5′-GGCTGTATTCCCCTCCATCG-3′; Reverse, 5′-AGCACTGTGTTGGCGTACAG-3′ were used.

### 2.7 Immunoblotting

Hypothalamic homogenates were prepared in a RIPA buffer using a Precellys^®^ homogenizer and zirconium oxide beads (Bertin Instruments, France) as previously described [21]. Membranes were incubated with rabbit anti-mouse CB_1_R (Frontier Institute Co, Ltd, Japan) and β-actin (Abcam) antibodies.

### 2.8 Blood biochemistry

Serum levels of cholesterol, high-density lipoprotein (HDL), low-density lipoprotein (LDL), alanine aminotransferase (ALT), and aspartate aminotransferase (AST) were determined using the Cobas C-111 chemistry analyzer (Roche, Switzerland). Serum insulin and leptin levels were measured by ELISA kits (Insulin, Crystal Chem, Inc., Downers Grove, IL, USA; Leptin, B-Bridge International, Santa Clara, CA, USA). Fasting blood glucose was measured by the Elite glucometer (Bayer, Pittsburgh, PA).

### 2.9 Glucose tolerance test (ipGTT), insulin resistance, and insulin sensitivity index

Mice that fasted overnight were injected with glucose (1.5 g/kg, i.p.), followed by a tail blood collection at 0, 15, 30, 45, 60, 90, and 120 min. Blood glucose levels were determined using the Elite glucometer (Bayer, Pittsburgh, PA). HOMA-IR was calculated as described above for humans. Fasting glucose and insulin levels were used to calculate the relative insulin sensitivity index (ISI) as 1/(glucose × insulin) × 1000, with glucose expressed as mg/dL and insulin as mU/L.

### 2.10 Hepatic triglyceride (TG) content

Liver tissues were extracted as described previously [21], and its TG content was determined using the EnzyChromTM TG Assay Kit (BioAssay Systems).

### 2.11 Materials

JD5037 and SLV319 were synthesized as described previously [26].

### 2.12 Statistics

#### 2.12.1 Humans

Statistical analyses were performed separately for each cohort using the IBM SPSS 22.0 software. Skewed data were normalized by log transformation. To test differences in continuous variables between PWS and normal healthy controls, independent samples t-tests were performed. ANCOVAs adjusting for age, sex, race, and BMI (BMI-Z for the American cohort of children) were also performed. Spearman correlation was used to test the relations between eCBs and metabolic parameters in the Israeli cohort. Nominal significance was set at *P* < 0.05, with Bonferroni correction for multiple comparisons (3 primary outcome measures: AEA, 2-AG, and AA, *P* < 0.017, and 14 measures for the correlation analysis with metabolic parameters, *P* < 0.003).

#### 2.12.2 Animals

Values are expressed as mean ± SEM. Unpaired two-tailed Student's t-test was used to determine differences between groups. Results in multiple groups and time-dependent variables were compared by ANOVA, followed by a Bonferroni test (GraphPad Prism v6 for Windows). Significance was set at *P* < 0.05.

## 3 Results

### 3.1 Increased circulating eCBs in patients with PWS

**Figure 1:**
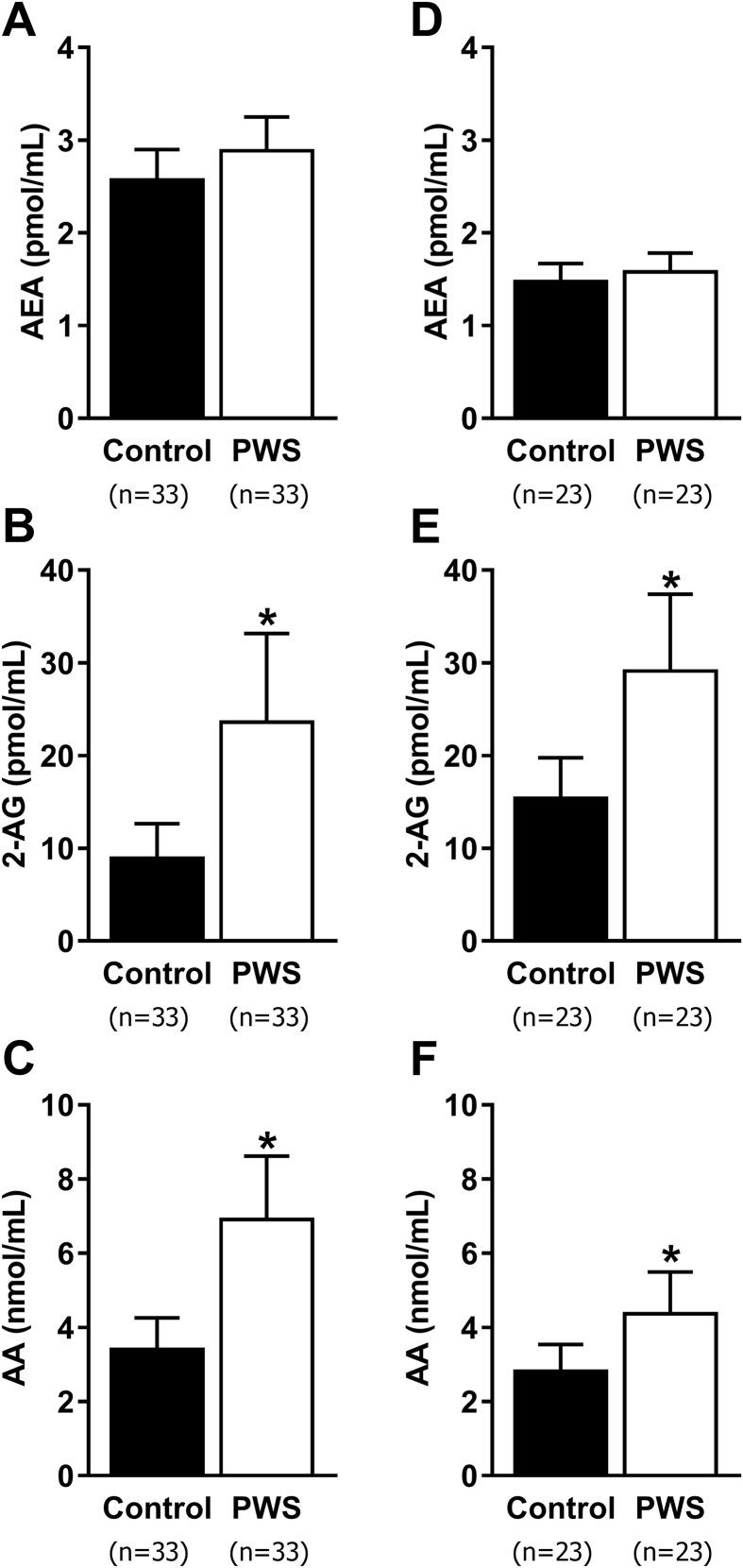
**Circulating eCB levels in patients with PWS compared with healthy controls, in two different cohorts**. Plasma levels of AEA (**A**, **D**), 2-AG (**B**, **E**), and AA (**C**, **F**), adjusted for age, sex, BMI (Israeli; **A**–**C**), or BMI-Z (North American; **D**–**F**), and race (North American; **D**–**F**) are shown as back-transformed adjusted mean ± 95% confidence interval in two different cohorts. *P < 0.05 relative to healthy controls of the same cohort.

To assess how the eCB system contributes to the development of obesity in PWS, we measured the plasma levels of AEA, 2-AG, and their endogenous precursor and breakdown ligand, AA, in two independent cohorts of patients with PWS. Circulating eCBs were determined in adults with PWS from Israel, and infants, children, and adults with PWS from North America in comparison with their healthy controls that were matched by the age, sex, BMI, and BMI-Z. Patient characteristics and comparisons are shown in Table 1. After correction for age, sex, BMI (Israeli) or BMI-Z (North American), and race (North American), adjusted 2-AG levels were significantly higher in PWS compared to controls in both the Israeli (*P* = 0.005) and North American (*P* = 0.002) clinical cohorts. The Israeli PWS patients also had significantly higher (*P* < 0.001) plasma concentrations of adjusted AA, while in the North American cohort, adjusted AA was nominally higher (*P* = 0.02) (Figure 1A–F). When African–American subjects, who comprised 52% of control subjects but none of the PWS patients, were excluded from the North American cohort analysis, AA was nominally higher (median [25th–50th percentile]; *P* = 0.03) in PWS (4.1 [2.5–5.6]; n = 23) vs. controls (2.8 [2.2–3.2]; n = 11) and remained so after adjustment for age, sex, and BMI-Z (*P* = 0.04). Next, we evaluated the correlations between circulating eCBs and metabolic parameters in the Israeli PWS cohort, and found that 2-AG was nominally negatively correlated with serum HDL levels (r = −0.51, *P* < 0.02), and AA was nominally positively correlated with HOMA-IR (r = 0.49; *P* < 0.03). Although adjusted AEA levels were comparable between the groups, it was nominally correlated with age (r = −0.41; *P* < 0.02) in subjects with PWS but not within control subjects (Supplementary Table 1).

### 3.2 Disrupted body composition and energy profiles in *Magel2*-null mice

**Figure 2:**
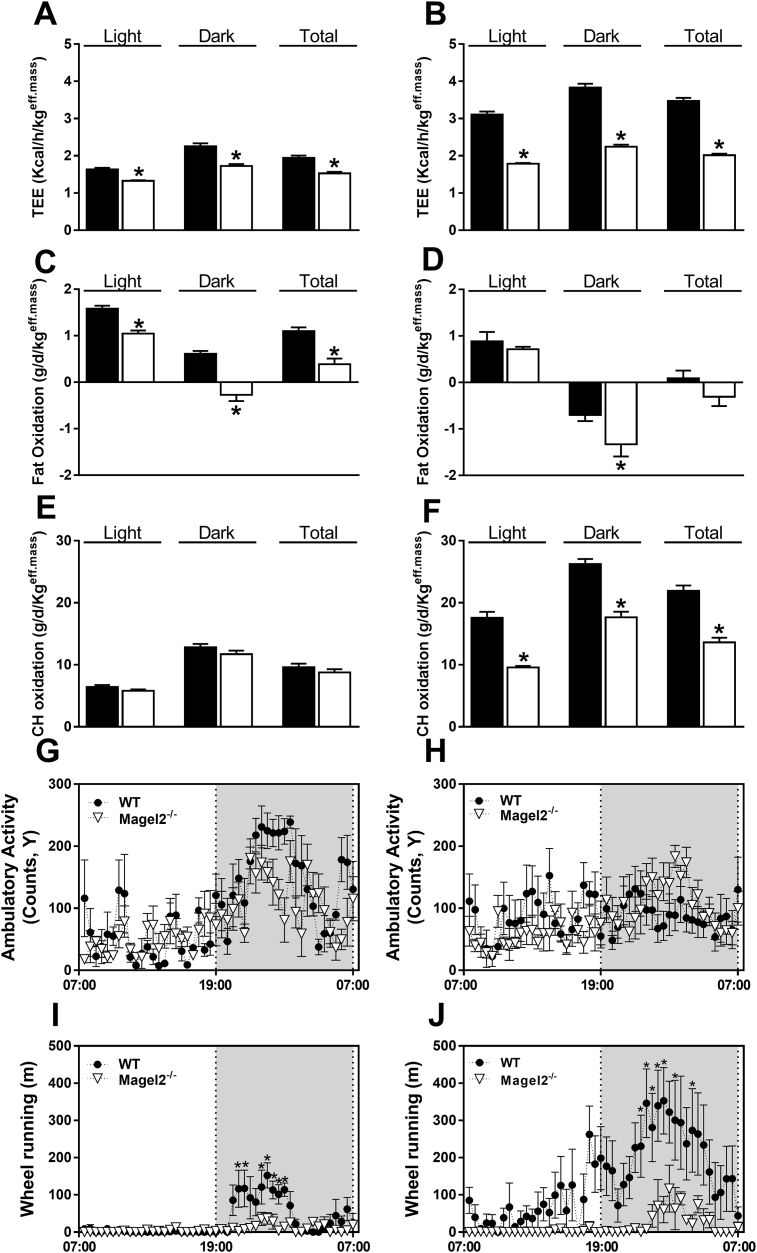
**Disrupted energy balance in *Magel2*-null mice**. As measured by indirect calorimetry over a 24 h period at 21 weeks of age, STD-fed *Magel2*-null mice exhibited abnormalities in TEE (**A**, **B**), FO (**C**, **D**), and CHO (**E**, **F**). These changes were not related to their ambulatory activity (**G**, **H**), but to their ability to run on a wheel, a corresponding measurement of voluntary activity (**I**, **J**). Similar patterns of results were obtained both in male (*left panels*) and female (*right panels*) mice. Legend: black bars, wild-type; white bars, *Magel2-null* mice. Data represent the mean ± SEM from 5 to 15 mice per group. *P < 0.05 relative to wild-type controls of the same sex.

To further delineate how the eCB/CB_1_R system is involved in developing obesity in PWS, we extended our studies to mice. Among the different gene mutations involved in PWS that have been engineered into mice (such as SNORD116/MBII-85, makorin-3 (*Mkrn3*), and necdin (*Ndn*) [27]), only *Magel2*-null mice recapitulate some of the metabolic and hormonal aspects of humans with PWS [22], [24], [28]. Whereas male *Magel2*-null mice exhibited a similar body weight gain curve from 6 to 18 weeks of age compared with their littermate wild-type controls, a significant increase in body weight was observed over time in female *Magel2*-null mice (Supplementary Figure 2A,B). Altered body composition, associated with increased fat mass (Supplementary Figure 2C,D) and no changes in lean body mass (Supplementary Figure 2E,F), was observed every four weeks (data not shown) and at 18 weeks of age in both male and female *Magel2*-null mice. In addition, the *Magel2*-null mice exhibited reduced TEE (Figure 2A,B), and FO (Figure 2C,D) in both sexes, while CHO was only reduced in female null mice (Figure 2E,F). Interestingly, whereas ambulatory activity did not differ appreciably between the two genotypes (Figure 2G,H), both male and female *Magel2*-null mice exhibited a significant reduction in voluntary activity, as measured by their ability to run on a wheel during the time that they spent in the metabolic chambers (Figure 2I,J). Taken together, these findings may explain the underlying metabolic mechanism by which *Magel2*-null mice gain more fat.

### 3.3 Increased hypothalamic, but not central or peripheral eCB ‘tone’ in *Magel2*-null mice

**Figure 3:**
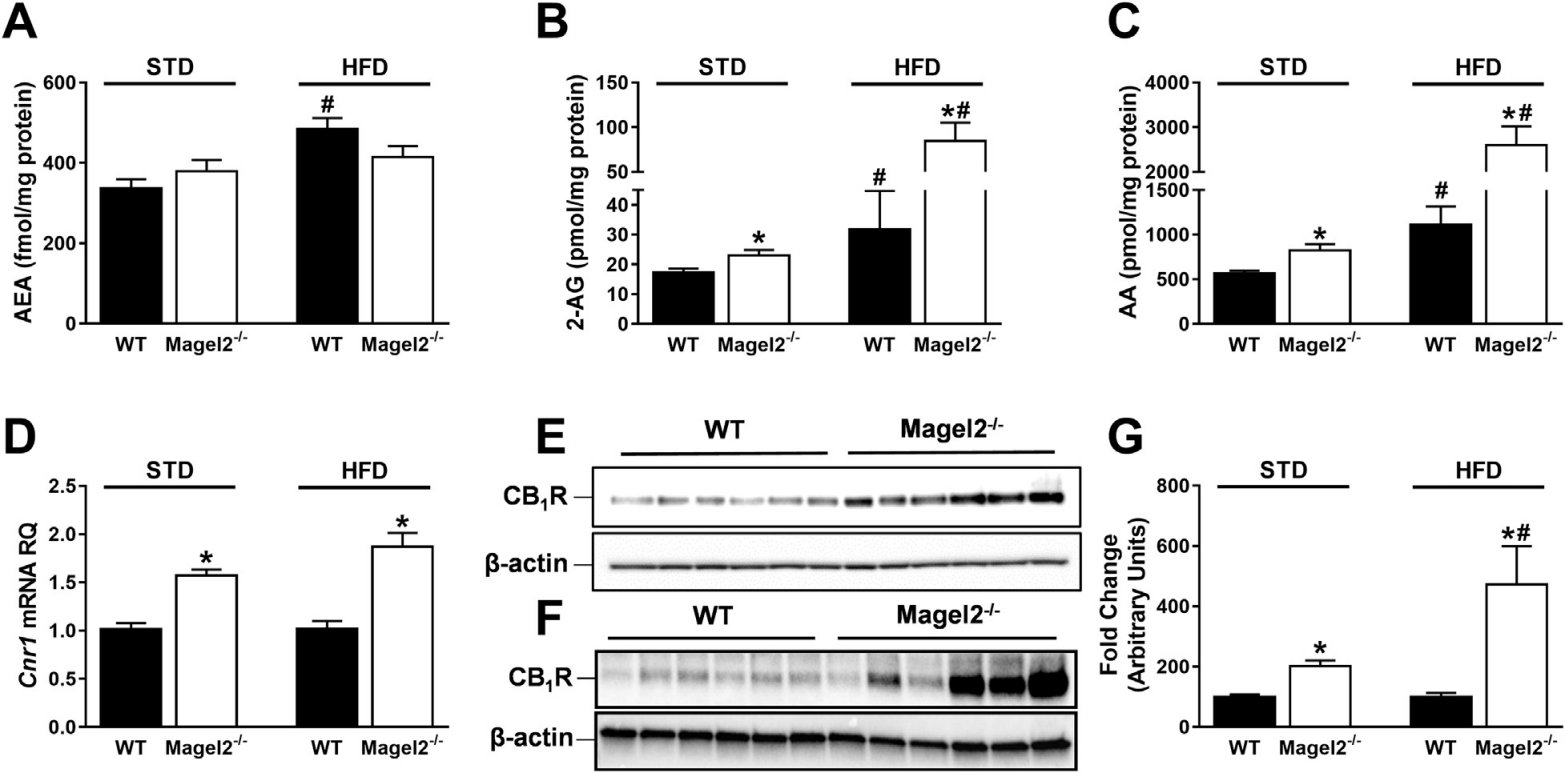
**Increased hypothalamic eCB ‘tone’ in *Magel2*-null *mice***. Hypothalamic AEA (**A**), 2-AG (**B**), and AA (**C**), levels in *Magel2*-null mice and littermate controls fed either STD (*left*) or HFD (*right*). A significant upregulation in the mRNA (**D**), and protein (**E**–**G**) expression levels of CB_1_R were measured in *Magel2*-null mice under both diets (STD, **D**, **E**, **G**; HFD, **D**, **F**, **G**), suggesting an increased eCB ‘tone’ in the hypothalamus. Data represent the mean ± SEM from 12 to 21 mice per group. *P < 0.05 relative to wild-type controls of the same diet. ^#^*P* < 0.05 relative to the same genotype on STD.

Unlike our findings in subjects with PWS, we did not detect differences in the circulating levels of 2-AG, AEA, and AA between the two mouse genotypes in both sexes, fed either a STD or a HFD (Supplementary Table 2–5). Moreover, *Magel2*-null mice had a similar amount of eCBs and an identical CB_1_R expression as their wild-type controls in the cerebral cortex, epididymal, retroperitoneal, and subcutaneous fat pads on either diet (Supplementary Table 2–5). However, analyzing the eCB ‘tone’ in the hypothalamus, the primary region of the brain involved in feeding, energy metabolism, and the area where *Magel2* is most highly expressed [29] revealed no changes in AEA levels (Figure 3A), yet a significant increase in 2-AG (Figure 3B) and AA (Figure 3C) levels. Moreover, we found a profound and significant upregulation in the gene (Figure 3D) and protein expression (Figure 3E–G) levels of CB_1_R. These findings were documented in mice fed either a STD or a HFD. Altogether, these results suggest that the hypothalamic eCB system plays a significant role in the altered metabolic profile identified in *Magel2*-null mice.

### 3.4 Peripherally restricted CB_1_R blockade reverses obesity and its metabolic abnormalities in *Magel2*-null mice

As with global CB_1_R knockout mice [30], [31], animals with a selective genetic deletion [17] or downregulation of CB_1_R in CaMKIIa-containing neurons [32] are lean and resistant to HFD-induced obesity. These findings suggest that CB_1_R in the hypothalamus is required for the development of obesity. However, this does not negate the role of peripheral CB_1_R in reversing obesity, since a globally acting CB_1_R blockade reduces body weight in obese but not in lean mice [33], and peripherally restricted CB_1_R antagonists ameliorate obesity and its related metabolic abnormalities in DIO mice [20], [21]. Therefore, we decided to test the efficacy of the peripherally restricted CB_1_R antagonist, JD5037, in treating obesity in *Magel2*-null mice. To further augment their obese phenotype, we fed both genotypes with a HFD for 12 weeks, then began daily treatment with JD5037 (3 mg/kg/d, i.p.) for 28 days. Males and females of both genotypes developed obesity under HFD conditions (Supplementary Figure 2G,H), with increased fat mass (Supplementary Figure 2I,J) and no major changes in lean body mass (Supplementary Figure 2K,L). As with *Magel2*-null mice on a STD (Supplementary Figure 2A,B), body weight gain was more pronounced in female knockout animals (Supplementary Figure 2G,H).

**Figure 4:**
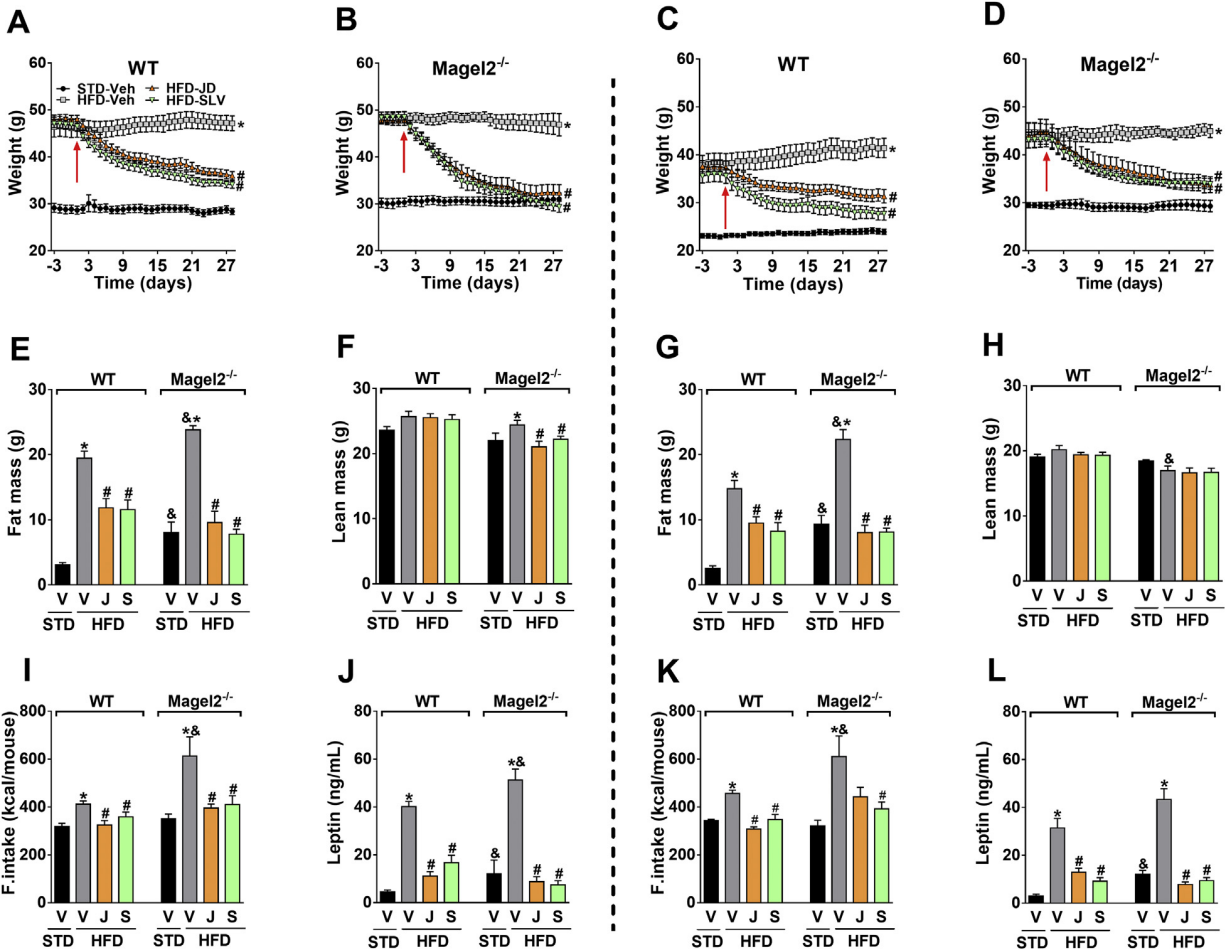
**Peripherally restricted CB**_**1**_**R antagonism reduces body weight, hyperphagia, and adiposity in *Magel2*-null mice**. Both JD5037 and SLV319 (3 mg/kg/day for 28 d) reduced body weight (**A**–**D**), and fat mass (**E**, **G**), without affecting lean body mass (**F**, **H**). These effects were associated with normalizing food intake (**I**, **K**), and serum leptin levels (**J**, **L**) in HFD-induced obese *Magel2*-null mice. Similar patterns of results were obtained both in male (*left panels*) and female (*right panels*) mice. Legend: Vehicle, V; JD5037, J; SLV319, S.; red arrow represents 1st day of treatment. Data represent the mean ± SEM from 5 to 10 mice per group. *P < 0.05 relative to STD-V of the same genotype; ^#^*P* < 0.05 relative to HFD-V of the same genotype; ^&^*P* < 0.05 relative to the same treatment group of the other genotype.

**Figure 5:**
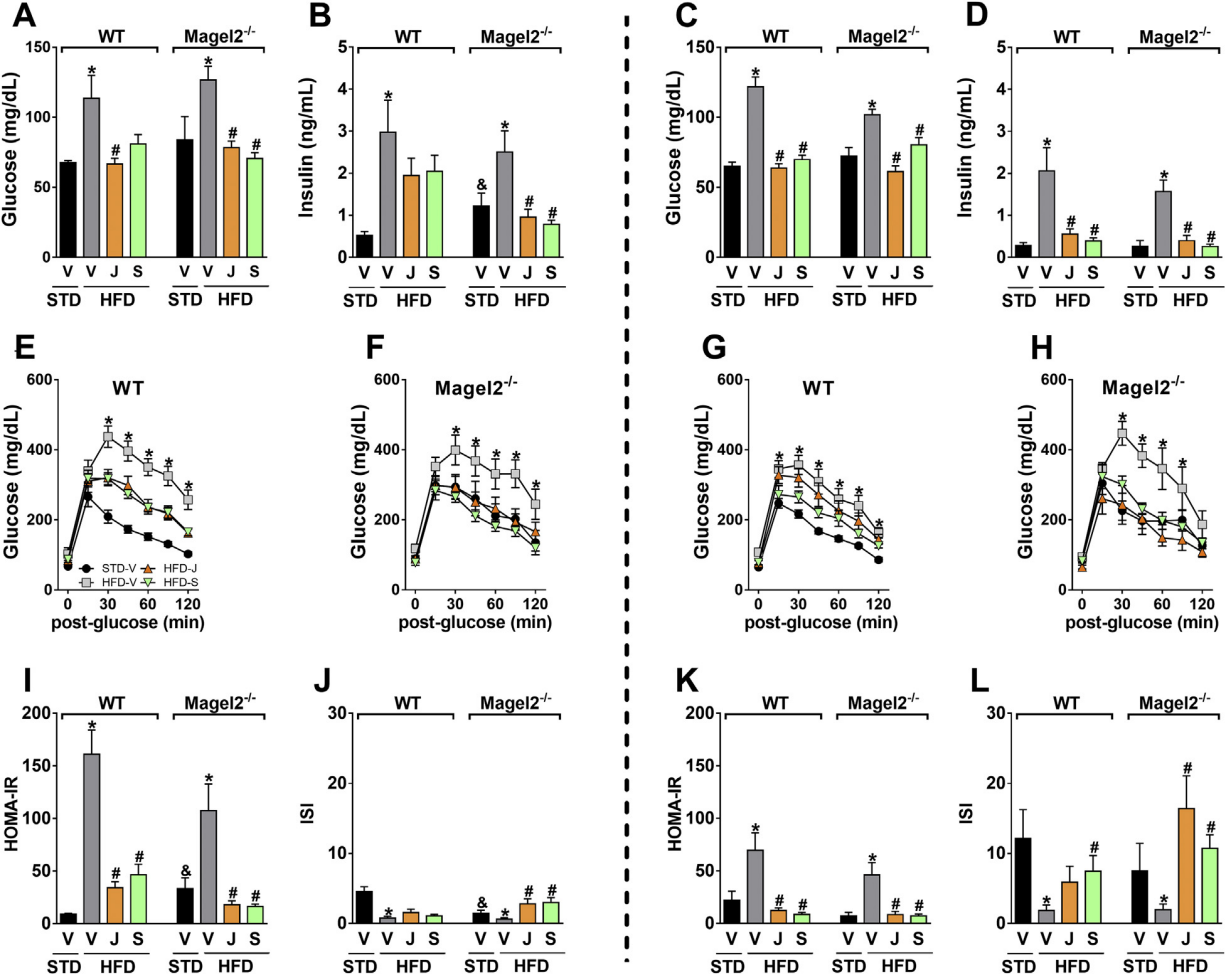
**Peripherally restricted CB**_**1**_**R antagonism improves glucose homeostasis in obese *Magel2*-null mice**. JD5037 and SLV319 (3 mg/kg/day for 28 d) attenuated the HFD-induced hyperglycemia (**A**, **C**), hyperinsulinemia (**B**, **D**), glucose intolerance (**E**–**H**), HOMA-IR (**I**, **K**), and insulin sensitivity index (**J**, **L**) in both obese male (*left panels*) and female (*right panels*) *Magel2*-null mice. Legend: Vehicle, V; JD5037, J; SLV319, S. Data represent the mean ± SEM from 5 to 10 mice per group. *P < 0.05 relative to STD-V of the same genotype; ^#^*P* < 0.05 relative to HFD-V of the same genotype; ^&^*P* < 0.05 relative to the same treatment group of the other genotype.

Since the globally acting CB_1_R antagonist rimonabant reduced body weight and improved metabolic features in humans with PWS, we compared the effect of JD5037 to its brain-penetrant parent compound, SLV319. Both JD5037 and SLV319 induced equal reductions in body weight (Figure 4A–D) and fat mass (Figure 4E,G). No major changes were documented in lean body mass (Figure 4F,H) in both genotypes and sexes. HFD-fed *Magel2*-null mice were hyperphagic, consuming significantly larger amounts of food in comparison with their wild-type littermates (Figure 4I,K). Both antagonists induced equal reductions in food consumption. Normalization of body weight in *Magel2*-null mice treated with JD5037 and SLV319 was probably due to the reversal of the hyperleptinemia (Figure 4J,L) and consequently improved leptin sensitivity, and not because of increased brain penetrance of JD5037 in *Magel2*-null mice, as reflected by a brain/plasma concentration ratio of <2% (Supplementary Figure 3A,B) in both strains. The two compounds were also equally effective in normalizing blood glucose (Figure 5A,C) and serum insulin (Figure 5B,D) levels, as well as in attenuating glucose intolerance (Figure 5E–H) and insulin resistance, as measured by the HOMA-IR and insulin sensitivity indices (Figure 5I–L) in both mouse genotypes and sexes.

**Figure 6:**
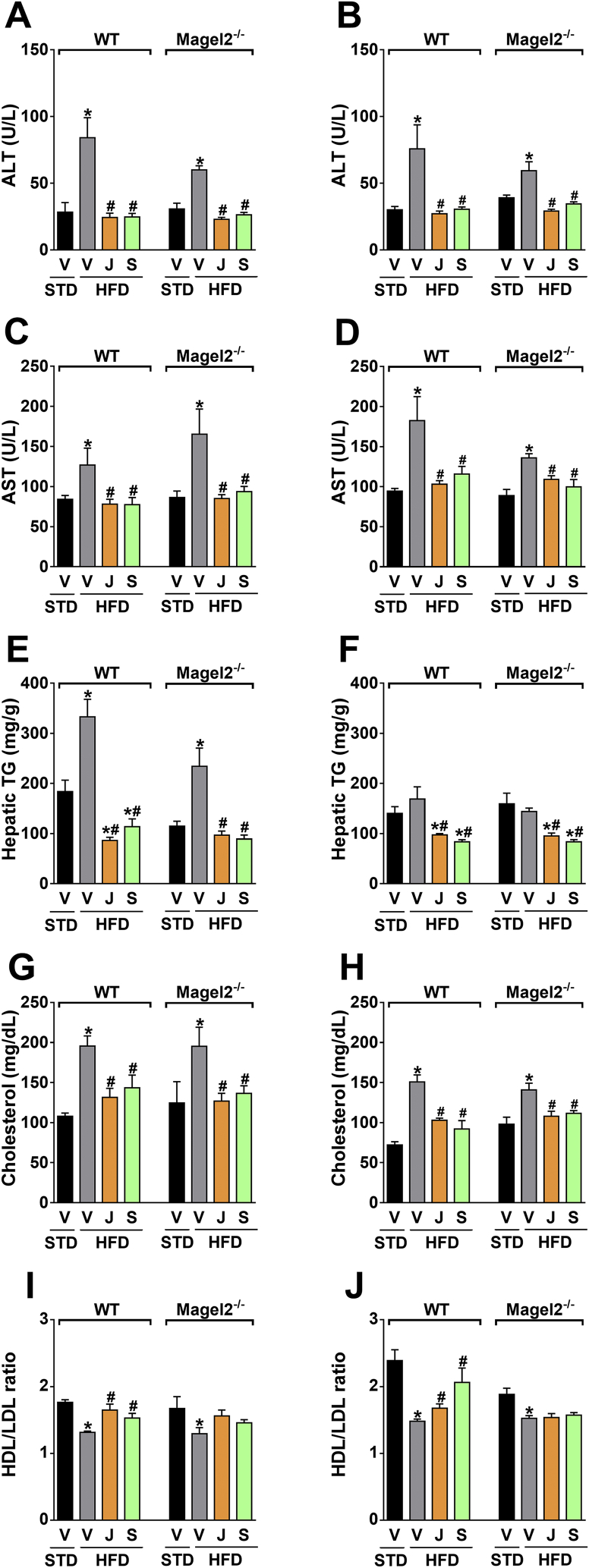
**Peripherally restricted CB**_**1**_**R antagonism restores liver function and cholesterol homeostasis in obese *Magel2*-null mice**. Both JD5037 and SLV319 (3 mg/kg/day for 28 d) treatment reduced the HFD-induced hepatic injury and steatosis in obese *Magel2*-null mice, as manifested by the reduced serum levels of ALT (**A**, **B**), AST (**C**, **D**), and TG content in the liver (**E**, **F**). Both compounds were equally potent in normalizing serum cholesterol levels (**G**, **H**), but not in restoring the HDL/LDL cholesterol ratio in *Magel2*-null mice (**I**, **J**). Similar patterns of results were obtained both in obese male (*left panels*) and female (*right panels*) mice. Legend: Vehicle, V; JD5037, J; SLV319, S. Data represent the mean ± SEM from 5 to 10 mice per group. *P < 0.05 relative to STD-V of the same genotype; ^#^*P* < 0.05 relative to HFD-V of the same genotype.

**Figure 7:**
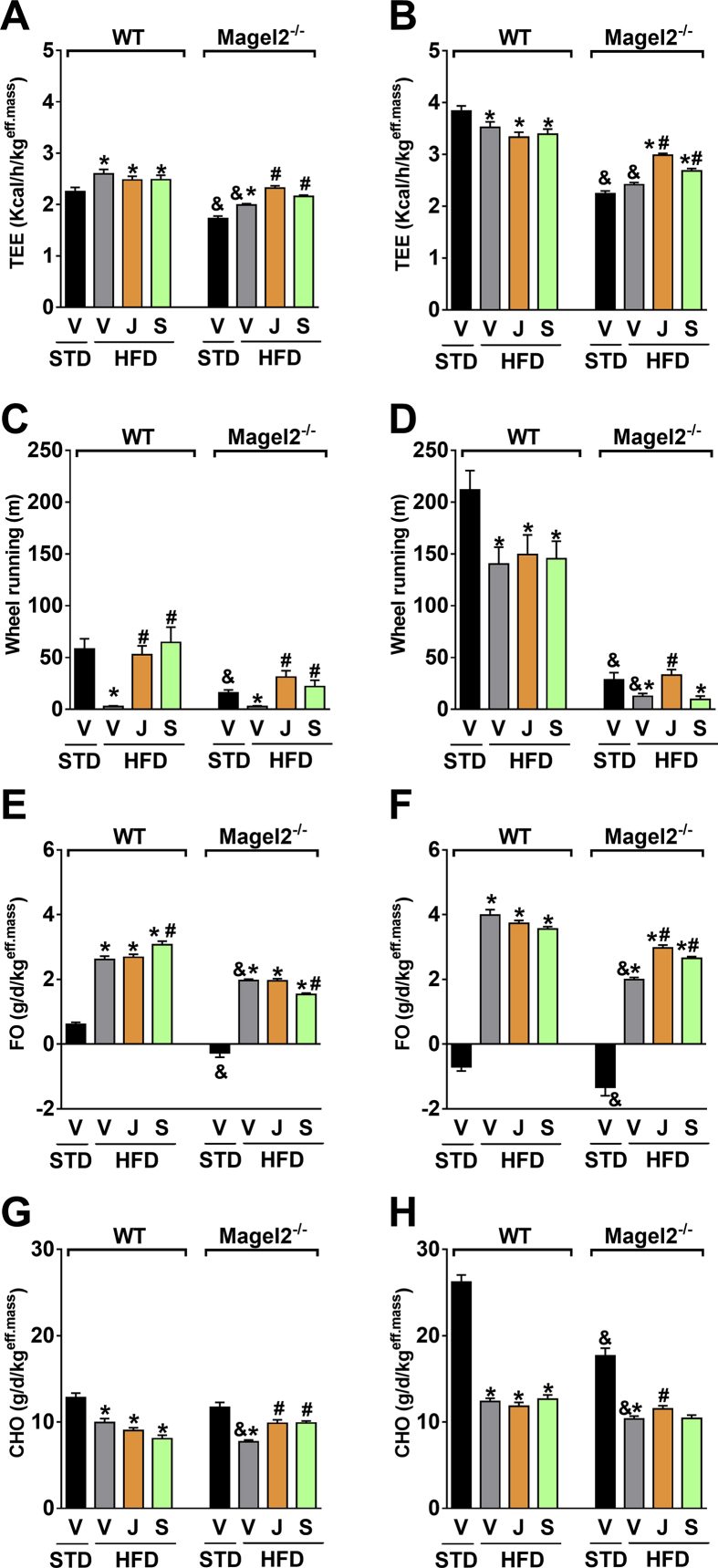
**Peripherally restricted CB**_**1**_**R antagonism restores the energy profile in obese *Magel2*-null mice**. Daily chronic treatment of HFD-induced obese *Magel2*-null mice and their littermate wild-type controls with JD5037 and SLV319 (3 mg/kg/day for 28 d) increases TEE (**A**, **B**), voluntary activity (**C**, **D**), FO (**E**, **F**), and CHO (**G**, **H**), as measured by indirect calorimetry. Almost similar patterns of results were obtained both in obese male (*left panels*) and female (*right panels*) mice. Legend: Vehicle, V; JD5037, J; SLV319, S. Data represent the mean ± SEM from 8 to 10 mice per group. *P < 0.05 relative to STD-V of the same genotype; ^#^*P* < 0.05 relative to HFD-V of the same genotype; ^&^*P* < 0.05 relative to the same treatment group of the other genotype.

Equal improvements were found in the ability of the peripherally restricted CB_1_R blockade to reverse hepatocellular damage and hepatic steatosis, as manifested by reduced serum levels of ALT (Figure 6A,B), AST (Figure 6C,D), and hepatic TG content (Figure 6E,F), as well as a reduction in the elevated serum cholesterol levels (Figure 6G,H). As shown previously [21], JD5037 accumulated in the liver in both strains (Supplementary Figure 3C), an effect that may account for its high efficacy in reversing hepatic steatosis. Interestingly, neither compound normalized the HDL-to-LDL cholesterol ratio in *Magel2*-null mice (Figure 6I,J). Indirect calorimetry in male and female *Magel2*-null mice and their littermate controls revealed a similar increase in TEE (Figure 7A,B) voluntary activity (Figure 7C,D), FO (Figure 7E,F), and CHO (Figure 7G,H) in animals treated with JD5037. Taken together, these findings suggest that blocking CB_1_R at the periphery reverses obesity, reduces hyperphagia, and improves metabolic outcomes in obese *Magel2*-null mice.

## 4 Discussion

Complications of severe obesity in PWS result in the high morbidity and mortality observed in this population. Currently, management of obesity in PWS is limited to maintaining a strict regimen with calorie restriction and daily physical activity. Although growth hormone is beneficial in reducing fat mass and increasing lean body mass, to date, there are no safe and effective medications for treating obesity in PWS. Endogenous and exogenous CBs are known to increase appetite and to promote obesity mainly through their interactions with the hypothalamic feeding circuitry [3], and CB_1_R antagonists were found effective in reducing body weight in experimental animals and in individuals with the common form of diet-related obesity [8], [9], [11]. Interestingly, it was recently reported that the CB_1_R antagonist rimonabant has the potential to improve metabolic profiles in adults with PWS [12]. However, further clinical testing of rimonabant and other globally acting CB_1_R antagonists was halted due to neuropsychiatric side effects mediated by the blockade of CB_1_Rs in the CNS [34], [35]. The present study is the first to provide evidence that in humans, circulating levels of eCBs are markedly elevated in PWS and appear to be correlated with several metabolic parameters. Moreover, our study demonstrates that (i) chronic blockade of CB_1_R in the periphery is as effective as globally acting CB_1_R antagonism in reversing obesity and its metabolic abnormalities in an established mouse model for PWS, and (ii) the disrupted body composition and energy balance in *Magel2*-null mice is associated with increased eCB ‘tone’ in the hypothalamus. Thus, targeting the eCB/CB_1_R system may represent a novel approach to treat obesity and its metabolic complications in PWS.

Circulating eCBs have been implicated in the pathogenesis of obesity and the metabolic syndrome both in humans and in animal models [36]. Here, we show, for the first time, that the circulating levels of 2-AG and its endogenous precursor ligand and breakdown product AA are markedly elevated in individuals with PWS. Moreover, 2-AG levels are negatively correlated with plasma HDL, whereas circulating AA is positively correlated with HOMA-IR, suggesting that they may play a contributing role in lipid metabolism and insulin homeostasis during obesity. These findings are consistent with findings in men with obesity, in whom 2-AG levels in the blood, but not AEA, correlated positively with BMI, adiposity, TGs, and insulin resistance, and negatively with HDL and adiponectin levels [37]. Similar findings also reported a positive correlation between circulating 2-AG levels and the degree of visceral adipose tissue content, TGs, and HDL [38]. Although the source of circulating 2-AG has not yet been documented, a possible mechanism underlying its elevated levels in PWS may lie in the upregulated activity of its bio-synthesizing enzymes, diacylglycerol lipase alpha and beta [3], and not an increased ingestion of a fatty diet since these PWS subjects are normally maintained on a healthy calorie-restricted diet to prevent weight gain. As per the increased amounts of circulating AA that was also documented here in subjects with PWS, a possible mechanism that may underlie this observation could be the increased rate in the metabolism of 2-AG to its component endogenous degrading products, AA and glycerol. 2-AG can be metabolized by different serine hydrolases, such as monoacylglycerol lipase, fatty acid amide hydrolase, as well as α-β-hydrolase domain 6 and 12 enzymes [39].

In the current study, we included two cohorts of PWS patients who exhibited similar elevations in plasma 2-AG levels but not AA. The main differences between the two cohorts were the age of the subjects and the absence of growth hormone therapy in the Israeli PWS group, which together may have affected the circulating levels of AA. One major limitation of our study is that although the subjects with PWS in both cohorts were predominantly Caucasians, the North American healthy matched controls included 52% African Americans, who generally have higher plasma levels of AA compared with Caucasians, due to genetic variants (SNP rs174537) in the fatty acid desaturase enzyme that converts linoleic acid to AA [40]. Because inclusion of race as a covariate or exclusion of African American subjects in the analysis yielded nominally higher AA levels in PWS, we could predict that had only Caucasian healthy subjects been included in the North American control cohort, a significant elevation in AA levels in PWS patients would have also been observed.

In contrast to the abnormal circulating eCB levels in subjects with PWS, we could not discern any major changes in eCB ‘tone’ in the serum, brain, and fat pads of *Magel2*-null mice. This suggests that other genes inactivated in PWS may also contribute to dysregulation of the eCB system, or may point to differences between species in the regulation of the eCB system. Nevertheless, our findings highlight *Magel2* as a key regulator of eCB ‘tone’ in the hypothalamus, where the gene is mostly expressed [29]. As previously reported [22], [24], [41], our data show that *Magel2*-null mice have increased adiposity and reduced energy expenditure but not increased food intake on a STD, with reduced FO, CHO, and voluntary activity. On a HFD, they become hyperphagic, hypoactive, and even more obese. Since activation of hypothalamic CB_1_R has been shown to modulate feeding, energy balance, and activity [42], [43], [44], [45], [46], our findings of upregulation of hypothalamic eCB ‘tone’ in *Magel2*-null mice fed either a STD or a HFD may further provide evidence that the eCB system plays a direct and critical role in developing hyperphagia and obesity as well as in altering the metabolic profile in PWS. These findings are in full agreement with several reports documenting increased hypothalamic 2-AG levels in the hyperphagic and obese *ob/ob* and *db/db* mice and Zucker (*fa/fa*) rats [3], as well as in the HFD-induced obese mice [47]. As suggested for exogenous cannabinoids [44], the possible molecular mechanism underlying the hyperphagia in *Magel2*-null mice could be the overactivation of CB_1_R specifically in POMC neurons, which, in turn, may lead to increased levels of the appetite regulator β-endorphin. In fact, the circulating levels of β-endorphin recently were found to be upregulated in subjects with PWS and were suggested to contribute to their hyperphagia [48]. Having said that, the central nervous system clearly utilizes a complex neuronal network that involves inter-organ communication via bidirectional pathways between the hypothalamus and several peripheral tissues [49], [50]. Therefore, the activity in a specific hypothalamic circuit can be controlled not only centrally, but also by peripheral organs. This is particularly important when one reflects on the regulation of energy metabolism via CB_1_R receptors, which are not only abundant in the brain, but also exist at much lower yet functionally relevant levels in various peripheral organs, including adipose tissue, skeletal muscle, liver, pancreas, kidney, and bone [51], as well as in peripheral sympathetic [52], [53] and parasympathetic terminals [54] and sensory neurons in the periphery [55].

If peripherally located CB_1_Rs do contribute to metabolic regulation, then restricting the penetrance of CB_1_R antagonists to the brain may improve their therapeutic index by reducing/eliminating the risk for neuropsychiatric side effects that were observed in obese subjects [34], [35] and in humans with PWS [12] treated with rimonabant. Our findings that the peripherally restricted CB_1_R antagonist JD5037 is as effective as its globally acting, brain-penetrant parent compound, SLV319 (ibipinabant), in ameliorating metabolic abnormalities in hyperphagic, obese *Magel2*-null mice clearly suggest that blocking CB_1_R in the periphery, rather than centrally, has potential efficacy for treating obesity in PWS.

The anorectic and weight-reducing effects of JD5037 have been already documented in DIO mice; it is suggested that they are mediated by increased central leptin sensitivity due to the reversal of HFD-induced hyperleptinemia [21]. Similar findings were also found here in obese *Magel2*-null mice, in which the marked increases in food intake and body weight during HFD feeding were associated with higher circulating levels of leptin, effects that were completely normalized when the mice were treated with JD5037. Although obesity in PWS was found to be unrelated to defective leptin production and/or a deficiency in the leptin receptor [56], [57], the hyperleptinemia found in adults with PWS, reflecting the increase in total adipose tissue mass, may in fact lead to the development of leptin resistance, as observed in the general obese population. Therefore, re-establishing leptin sensitivity by reducing the hyperleptinemia may represent a novel approach to treat obesity also in PWS. Indeed, the significant reductions in body weight, food intake, fat mass, and leptin levels in *Magel2*-null mice were as prominent as in their littermate, wild-type control animals, if not more. However, these findings are not in full agreement with the recent reports indicating that *Magel2*-null mice display central leptin resistance [58], [59]. On the other hand, Pravdivyi and colleagues have recently shown that young *Magel2*-null mice exhibit a normal hypothalamic response to leptin [60]. Therefore, the HFD feeding and its consequent hyperleptinemia could possibly trigger changes in hypothalamic leptin signaling circuits that then could be restored once leptin levels are reduced dramatically by a peripheral CB_1_R blockade. Moreover, reduced leptin sensitivity in *Magel2*-null mice was reported to be mediated via POMC neurons at the arcuate nucleus [58], [60]; thus, peripheral CB_1_R blockade-induced leptin sensitivity might be mediated via other neuronal/hypothalamic nuclei. Additionally, chronic treatment of *Magel2*-null mice with JD5037 was able to completely restore the HFD-induced reduction in TEE and voluntary activity and increase FO and CHO, a combined effect that could also explain the reduction in body weight. In fact, leptin is known to increase FO in adipose tissue and skeletal muscle [61], similar to the effect of CB_1_R antagonists in enhancing mitochondrial FO in the liver [15] and adipose tissue [62]. Therefore, increased sensitivity to the metabolic effects of endogenous leptin may also contribute to the increase in FO and consequently, weight loss following JD5037 treatment in *Magel2*-null mice. Nonetheless, we cannot exclude the possibility that other mechanisms (peripheral-central circuit) also may contribute to the effects of peripheral CB_1_R blockade on food intake and body weight.

Besides affecting body weight and food intake, JD5037 was found to be as efficacious as SLV319 in normalizing the elevated blood glucose and serum insulin levels, improving glucose tolerance and insulin resistance, reversing the HFD-induced hepatic steatosis, and improving the plasma lipid profile of obese *Magel2*-null mice. This suggests that these improved conditions are primarily due to the blockade of peripheral CB_1_R. Since CB_1_Rs are expressed in many tissues related to metabolic control [51], their possible contribution to the effects of JD5037 in *Magel2*-null mice remains to be explored.

Investigating both male and female rodents in a single pharmacology article is rare [63]; one of the main novelties of this work is that it is the first to report (i) a detailed analysis of eCB ‘tone’ in both male and female mice, and (ii) a complete metabolic profiling of both male and female HFD-induced obese *Magel2*-null mice. This study also compares the metabolic efficacy of blocking peripheral CB_1_Rs between two genotypes and across sexes. Interestingly, JD5037 had similar metabolic effects regardless of the sex and genotype of the treated animals. However, a higher sensitivity to insulin and lower levels of TGs in the liver under HFD conditions were two features that distinguished female *Magel2*-null mice from males. Nevertheless, a peripheral CB_1_R blockade was able to enhance these characteristics, suggesting that fluctuations in the female hormone status did not affect the pharmacokinetics and efficacy of JD5037.

In conclusion, the current study provides the first evidence that the eCB system may contribute to severe obesity both in PWS children and adults and in an established mouse model for this syndrome. Our results confirm that the eCB system contributes to the metabolic phenotype associated with PWS. In subjects with PWS, increased circulating eCBs were associated with their metabolic abnormalities, whereas in *Magel2*-null mice increased hypothalamic eCB ‘tone’, manifested by elevated tissue levels of CB_1_R and eCBs, may contribute to their altered metabolic profile. Moreover, specifically targeting the peripheral eCB system in obese *Magel2*-null mice was found to be as efficacious as in DIO animals, and, therefore, it may represent a novel approach to treating obesity and its complications in PWS. This would also provide the rationale for the development and clinical testing of peripherally restricted CB_1_R antagonists for treating obesity in PWS.

## Figures and Tables

**Table 1:** Clinical characteristics of the study population and comparison between subjects with and without PWS.

Israeli cohort	Control	PWS	*P* value
N	33	33	–
Age (yr)	28 ± 1.3	29 ± 1.4	n.s.
DEL/UPD/IC	–	19/13/1	–
Sex (% male)	50	50	n.s.
Race (% Caucasian)	100	100	n.s.
BMI (kg/m^2^)	28.9 ± 1.4	28.9 ± 1.4	n.s.
BMI-Z	1.2 ± 0.8	1.3 ± 0.8	n.s.
AEA (pmol/mL)	2.7 [2.1–3.3]	3.0 [2.3–3.4]	n.s.
2-AG (pmol/mL)	10.6 [4.4–22.8]	23.6 [13.0–38.2]	<0.001*
AA (nmol/mL)	3.2 [2.2–4.1]	7.5 [4.2–11.0]	<0.001*

N. American cohort	Control	PWS	*P* value
N	23	23	–
Age (yr)	13.1 ± 6.3	11.4 ± 7.7	n.s.
DEL/UPD/IC	–	14/8/1	–
Sex (% male)	61	61	n.s.
Race (% Caucasian)	35	78	0.001*
BMI (kg/m^2^)	24.8 ± 8.3	24.5 ± 10.0	n.s.
BMI-Z	1.2 ± 1.2	1.2 ± 1.2	n.s.
AEA (pmol/mL)	1.7 [1.2–1.9]	1.5 [1.2–1.8]	n.s.
2-AG (pmol/mL)	16.2 [12.4–17.9]	24.5 [16.6–42.0]	<0.001*
AA (nmol/mL)	3.2 [2.5–3.9]	4.1 [2.5–5.6]	n.s.

Data are expressed as mean ± SD for normally distributed data or median [interquartile rage] for skewed data. * *P* < 0.001. Not significant (n.s.).
