# Supplementary material for: Targeting the endocannabinoid/CB1 receptor system for treating obesity in Prader–Willi syndrome
Source: Mol Metab. 2016 Oct 22;5(12):1187–99. doi: 10.1016/j.molmet.2016.10.004 (PMC5123200; doi:10.1016/j.molmet.2016.10.004)
Supplement: Supplementary file 1 [file mmc1.pdf]

Targeting the Endocannabinoid/CB1 Receptor System for Treating Obesity in Prader-Willi Syndrome

Ibrahim Knani, Brian J. Earley, Shiran Udi, Alina Nemirovski, Rivka Hadar, Asaad Gammal, Resat Cinar, Harry J. Hirsch, Yehuda Pollak, Itai Gross, Talia Eldar-Geva, Daniella P. Reyes-Capo, Joan C. Han, Andrea M. Haqq, Varda Gross-Tsur, Rachel Wevrick, Joseph Tam

Supplementary Materials

Supplementary Fig. 1. Analysis of covariance (ANCOVA) for calculating metabolic data.

Supplementary Fig. 2. Altered body composition in *Magel2*-null mice fed a STD and a HFD.

Supplementary Fig. 3. Low levels of JD5037 in the brain in *Magel2*-null and littermate control mice.

Supplementary Table 1. Correlations among AEA, 2-AG, and AA and metabolic parameters in Israeli PWS patients.

Supplementary Table 2. eCB 'tone' in male *Magel2*-null mice and their littermate controls fed a STD.

Supplementary Table 3. eCB 'tone' in female *Magel2*-null mice and their littermate controls fed a STD.

Supplementary Table 4. eCB 'tone' in male *Magel2*-null mice and their littermate controls fed a HFD.

Supplementary Table 5. eCB 'tone' in female *Magel2*-null mice and their littermate controls fed a HFD.

Supplementary Fig. 1.

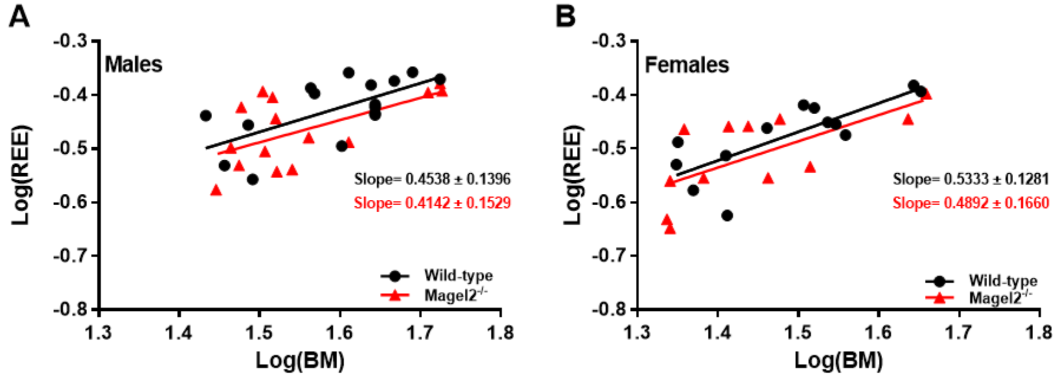

Analysis of covariance (ANCOVA) for calculating metabolic data. Logarithms of body mass and of the resting metabolic rate that were regressed against each other. The slops of the regression curves were used as the power to which body mass was raised in order to yield the panels describing TEE, FO and CHO in ***Figures 2 & 7***, in proportion to their metabolic rate.

Supplementary Fig. 2.

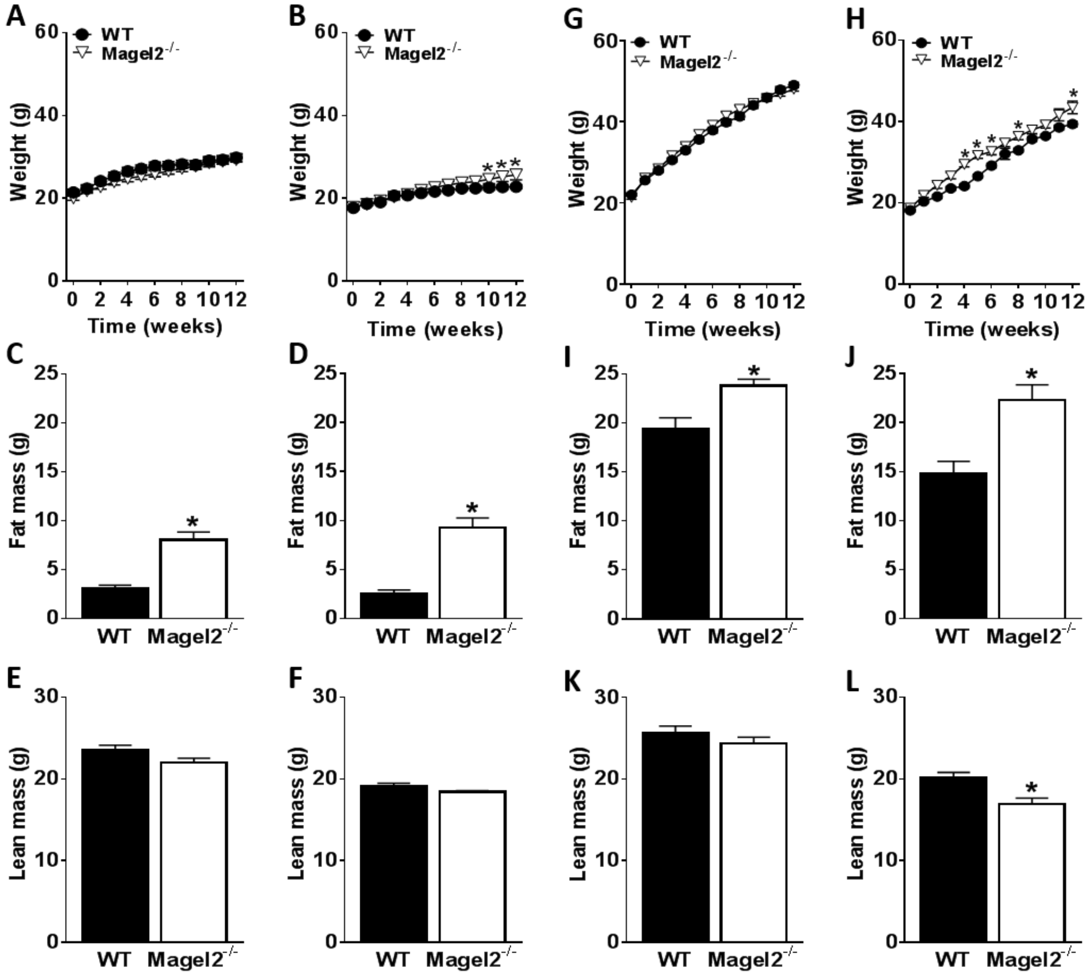

Altered body composition in *Magel2*-null mice fed either a STD or a HFD. *Magel2*-null mice and their littermate wild-type control animals were fed either a STD (**A-F**) or a HFD (**G-L**) for 12 weeks. Changes in body weight (**A, B, G, H**), fat mass (**C, D, I, J**), and lean mass (**E, F, K, L**) were measured in both male (**A, C, E, G, I, K**) and female (**B, D, F, H, J, L**) mice. Data represent the mean ± SEM from 15-23 mice per group. **P*<0.05 relative to wild-type controls of the same sex.

Supplementary Fig. 3.

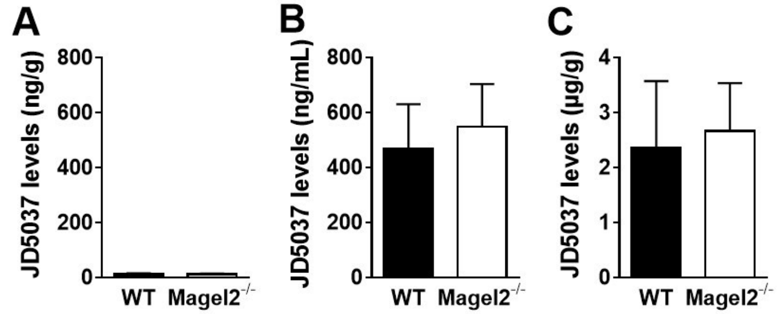

Low levels of JD5037 in the brain in *Magel2*-null and littermate control mice. Brain (**A**), serum (**B**), and liver (**C**) levels of JD5037 1 h following an acute administration at 3 mg/kg. Data represent the mean ± SEM from 3 mice per group. *P<0.05 relative wild-type controls.

Supplementary Table 1. Correlations among AEA, 2-AG, and AA and metabolic parameters in Israeli PWS patients

Parameter	AEA	2-AG	AA
**Age**	**-0.41***	-0.26	-0.21
**Weight**	-0.25	-0.05	0.09
**BMI**	-0.18	-0.05	0.05
**Insulin**	-0.01	-0.04	0.37
**Glucose**	-0.03	0.33	0.07
**Triglycerides**	0.06	0.36	0.23
**Cholesterol**	0.17	0.04	0.38
**HDL**	-0.13	**-0.51***	0.11
**LDL**	0.19	0.14	0.16
**CRP**	-0.12	0.10	0.00
**HOMA**	0.00	0.05	**0.49***
**Leptin**	0.10	-0.26	0.31
**Adiponectin**	-0.29	-0.33	-0.36

***** $P<0.05$

Supplementary Table 2. eCB 'tone' in male *Magel2*-null mice and their littermate controls fed a STD

		Wild-type	*Magel2*-null	*P* Value
**Blood**	AEA	1.5 ± 0.1	1.3 ± 0.1	n.s.
	2-AG	74.2 ± 6.1	63.5 ± 3.9	n.s.
	AA	6.0 ± 0.9	5.0 ± 0.7	n.s.
**Brain**	AEA	51.2 ± 5.3	83.8 ± 12.7	**0.04***
	2-AG	5.6 ± 0.6	4.3 ± 0.7	n.s.
	AA	6.2 ± 0.4	6.5 ± 0.5	n.s.
	Cnr1	1.0 ± 0.1	1.0 ± 0.1	n.s.
**Epi. Fat**	AEA	10.4 ± 0.5	11.8 ± 2.6	n.s.
	2-AG	1.4 ± 0.2	1.3 ± 0.1	n.s.
	AA	6.4 ± 0.8	3.3 ± 0.5	**0.01***
	Cnr1	1.1 ± 0.2	0.8 ± 0.1	n.s.
**RP. Fat**	AEA	5.8 ± 0.6	8.9 ± 1.5	n.s.
	2-AG	2.3 ± 0.4	1.7 ± 0.1	n.s.
	AA	14.6 ± 1.9	10.4 ± 0.4	n.s.
	Cnr1	1.1 ± 0.2	0.7 ± 0.1	n.s.
**SC. Fat**	AEA	10.0 ± 0.6	9.2 ± 0.7	n.s.
	2-AG	3.0 ± 0.4	2.5 ± 0.7	n.s.
	AA	14.9 ± 1.9	9.2 ± 0.4	**0.02***
	Cnr1	1.1 ± 0.2	0.7 ± 0.2	n.s.

Data represent the mean ± SEM from 4-5 animals per group. Values are expressed as pmol/mL or fmol/mg for AEA, pmol/mL or pmol/mg for 2-AG, and nmol/mL or pmol/mg for AA. *Cnr1* mRNA expression levels are expressed as relative values. Epididymal (Epi); Retroperitoneal (RP); Subcutaneous (SC); not significant (n.s.).

Supplementary Table 3. eCB 'tone' in female *Magel2*-null mice and their littermate controls fed a STD

		Wild-type	*Magel2*-null	*P* Value
**Blood**	AEA	7.4 ± 0.3	7.0 ± 0.4	n.s.
	2-AG	96.6 ± 11.8	85.4 ± 7.6	n.s.
	AA	11.6 ± 1.8	6.8 ± 1.0	n.s.
**Brain**	AEA	51.1 ± 8.0	60.8 ± 9.3	n.s.
	2-AG	6.9 ± 0.7	6.7 ± 0.6	n.s.
	AA	5.4 ± 0.4	3.7 ± 0.5	**0.04***
	Cnr1	1.2 ± 0.1	1.0 ± 0.2	n.s.
**Epi. Fat**	AEA	7.8 ± 0.2	6.9 ± 0.6	n.s.
	2-AG	1.6 ± 0.2	1.3 ± 0.2	n.s.
	AA	8.8 ± 0.4	5.3 ± 0.9	**0.01***
	Cnr1	1.0 ± 0.1	1.1 ± 0.2	n.s.
**RP. Fat**	AEA	16.2 ± 4.0	14.5 ± 4.2	n.s.
	2-AG	1.0 ± 0.1	1.0 ± 0.1	n.s.
	AA	8.2 ± 1.7	7.0 ± 1.7	n.s.
	Cnr1	1.1 ± 0.4	0.3 ± 0.1	n.s.
**SC. Fat**	AEA	10.0 ± 0.5	11.0 ± 1.8	n.s.
	2-AG	1.7 ± 0.2	1.5 ± 0.5	n.s.
	AA	15.2 ± 1.0	8.0 ± 1.5	**0.01***
	Cnr1	1.0 ± 0.1	0.6 ± 0.2	n.s.

Data represent the mean ± SEM from 4-5 animals per group. Values are expressed as pmol/mL or fmol/mg for AEA, pmol/mL or pmol/mg for 2-AG, and nmol/mL or pmol/mg for AA. *Cnr1* mRNA expression levels are expressed as relative values. Epididymal (Epi); Retroperitoneal (RP); Subcutaneous (SC); not significant (n.s.).

Supplementary Table 4. eCB 'tone' in male *Magel2*-null mice and their littermate controls fed a HFD

		Wild-type	*Magel2*-null	*P* Value
**Blood**	AEA	3.2 ± 0.1	3.5 ± 0.3	n.s.
	2-AG	67.7 ± 4.4	66.1 ± 5.7	n.s.
	AA	10.6 ± 0.7	12.7 ± 1.8	n.s.
**Brain**	AEA	55.6 ± 2.8	44.7 ± 11.9	n.s.
	2-AG	5.2 ± 0.4	5.3 ± 0.6	n.s.
	AA	6.2 ± 0.6	6.5 ± 0.4	n.s.
	Cnr1	1.0 ± 0.1	0.8 ± 0.1	n.s.
**Epi. Fat**	AEA	18.3 ± 1.7	14.2 ± 2	n.s.
	2-AG	3.5 ± 0.9	1.818 ± 0.7	n.s.
	AA	4.3 ± 0.3	4.5 ± 0.8	n.s.
	Cnr1	1.2 ± 0.3	1.0 ± 0.08	n.s.
**RP. Fat**	AEA	14.3 ± 1.7	11.1 ± 2.7	n.s.
	2-AG	1.9 ± 0.2	1.5 ± 0.4	n.s.
	AA	10.2 ± 1.1	8.8 ± 1.5	n.s.
	Cnr1	1.0 ± 0.1	1.0 ± 0.2	n.s.
**SC. Fat**	AEA	17.0 ± 2.2	13.4 ± 2.9	n.s.
	2-AG	1.5 ± 0.2	1.0 ± 0.2	n.s.
	AA	4.3 ± 0.7	7.8 ± 0.9	**0.01***
	Cnr1	1.1 ± 0.2	0.6 ± 0.2	n.s.

Data represent the mean ± SEM from 4-5 animals per group. Values are expressed as pmol/mL or fmol/mg for AEA, pmol/mL or pmol/mg for 2-AG, and nmol/mL or pmol/mg for AA. *Cnr1* mRNA expression levels are expressed as relative values. Epididymal (Epi); Retroperitoneal (RP); Subcutaneous (SC); not significant (n.s.).

Supplementary Table 5. eCB 'tone' in female *Magel2*-null mice and their littermate controls fed a HFD.

		Wild-type	*Magel2*-null	*P* Value
**Blood**	AEA	8.6 ± 0.2	9.3 ± 0.6	n.s.
	2-AG	107.3 ± 8.6	108.7 ± 7.7	n.s.
	AA	16.2 ± 2.3	12.7 ± 2.7	n.s.
**Brain**	AEA	31.2 ± 3.4	44.4 ± 2.6	**0.01***
	2-AG	6.9 ± 0.4	5.8 ± 0.3	n.s.
	AA	3.6 ± 0.5	4.3 ± 0.5	n.s.
	Cnr1	1.0 ± 0.1	1.2 ± 0.2	n.s.
**Epi. Fat**	AEA	14.7 ± 1.8	13.8 ± 0.2	n.s.
	2-AG	0.9 ± 0.1	0.9 ± 0.06	n.s.
	AA	6.1 ± 0.7	5.7 ± 0.7	n.s.
	Cnr1	1.1 ± 0.2	0.9 ± 0.1	n.s.
**RP. Fat**	AEA	15.0 ± 1.1	18.1 ± 1.4	n.s.
	2-AG	0.6 ± 0.1	0.8 ± 0.1	n.s.
	AA	7.9 ± 1.5	6.9 ± 0.7	n.s.
	Cnr1	1.2 ± 0.3	1.1 ± 0.4	n.s.
**SC. Fat**	AEA	19.8 ± 4.4	12.1 ± 2.5	n.s.
	2-AG	0.9 ± 0.1	0.9 ± 0.1	n.s.
	AA	9.6 ± 1.5	9.0 ± 1.1	n.s.
	Cnr1	1.1 ± 0.3	0.5 ± 0.2	n.s.

Data represent the mean ± SEM from 4-5 animals per group. Values are expressed as pmol/mL or fmol/mg for AEA, pmol/mL or pmol/mg for 2-AG, and nmol/mL or pmol/mg for AA. *Cnr1* mRNA expression levels are expressed as relative values. Epididymal (Epi); Retroperitoneal (RP); Subcutaneous (SC); not significant (n.s.).
